# Upper and lower limb tremor in Charcot–Marie–Tooth neuropathy type 1A and the implications for standing balance

**DOI:** 10.1007/s00415-023-12124-z

**Published:** 2023-12-05

**Authors:** Matthew Silsby, Con Yiannikas, Alessandro F. Fois, Marina L. Kennerson, Matthew C. Kiernan, Victor S. C. Fung, Steve Vucic

**Affiliations:** 1 Neurology Department, Westmead Hospital, Westmead Clinical School, University of Sydney, Sydney, NSW Australia; 2 Brain and Nerve Research Centre, University of Sydney, Concord, NSW Australia; 3 Neurology Department, Concord Hospital, Sydney, NSW Australia; 4 Neurology Department, Royal North Shore Hospital, Sydney, NSW Australia; 5 Northcott Neuroscience Laboratory, ANZAC Research Institute, University of Sydney, Sydney, NSW Australia; 6 Molecular Medicine Laboratory, Concord Hospital, Concord, NSW Australia; 7 Neurology Department, Royal Prince Alfred Hospital Sydney, Sydney, NSW Australia; 8 Brain and Mind Centre, University of Sydney, Camperdown, NSW Australia

**Keywords:** CMT1A, Neuropathy, Tremor, Balance

## Abstract

**Background:**

Neuropathic tremor occurs in Charcot–Marie–Tooth neuropathy type 1A (CMT1A; hereditary motor and sensory neuropathy, HMSN), although the pathophysiological mechanisms remain to be elucidated. Separately, lower limb tremor has not been explored in CMT1A and could be associated with imbalance as in other neuropathies. The present study aimed to determine tremor characteristics in the upper and lower limbs in CMT1A and relate these findings to clinical disability, particularly imbalance.

**Methods:**

Tremor and posturography studies were undertaken in phenotyped and genotyped CMT1A patients. Participants underwent detailed clinical assessment, tremor study recordings, and nerve conduction studies. Tremor stability index was calculated for upper limb tremor and compared to essential tremor.

**Results:**

Seventeen patients were enrolled. Postural and kinetic upper limb tremors were evident in 65%, while postural and orthostatic lower limb tremors were seen in 35% of CMT1A patients. Peak upper limb frequencies were lower distally (~ 6 Hz) and higher proximally (~ 9 Hz), were unchanged by weight-loading, and not impacted by fatigue. The tremor stability index was significantly higher in CMT1A than in essential tremor. A 5–6 Hz lower limb tremor was recorded which did not vary along the limb and was unaffected by fatigue. Balance was impaired in patients with postural lower limb tremor. A high frequency peak on posturography was associated with ‘good’ balance.

**Conclusions:**

Tremor is a common clinical feature in CMT1A, distinct from essential tremor, mediated by a complex interaction between peripheral and central mechanisms. Postural lower limb tremor is associated with imbalance; strategies aimed at tremor modulation could be of therapeutic utility.

**Supplementary Information:**

The online version contains supplementary material available at 10.1007/s00415-023-12124-z.

## Introduction

Neuropathic tremor is a recognized clinical feature of demyelinating neuropathies [1]. Charcot–Marie–Tooth (CMT) disease-type 1A is the commonest inherited neuropathy, characterized by diffuse demyelination and dysmyelination [2, 3]. Neuropathic tremor was described in a group of patients with Roussy–Levy Syndrome almost 100 years ago [4], a condition that is now recognized as consistent with Charcot–Marie–Tooth disease type 1 [5, 6]. Despite this long history, the pathophysiological mechanisms underlying the development of neuropathic tremor remain to be elucidated.

Previous work in patients with acquired inflammatory neuropathy suggests that disrupted peripheral nerve function confuses a central generator with resultant tremor [7–10]. In the case of CMT, a unifying hypothesis is lacking. CMT-tremor has been reported as indistinguishable from essential tremor (ET) both clinically [11] and neurophysiologically [12], particularly as the peak frequency of tremor in CMT falls within the same frequency band as ET [13]. This could suggest a shared pathophysiological mechanism, such as dysfunction of the cerebello–thalamo–cortical network [14]. Counter to this, a prior study in CMT1A argued against cerebellar dysfunction as a possible pathophysiological mechanism [15]. Separately, it has been argued that CMT-tremor may be related to muscle fatigue, causing an enhanced physiological tremor, as evident in healthy subjects [16, 17]. As such, detailed neurophysiological assessment, including recording of tremor stability index (TSI), may help to clarify the pathophysiological mechanisms of CMT-tremor.

Of further relevance, lower limb tremor in inherited neuropathies has not been previously assessed. A recent analysis of chronic inflammatory demyelinating polyradiculoneuropathy (CIDP) reported a lower limb tremor affected ~ 30% of patients and correlated with imbalance [18]. Given that imbalance is a prominent symptom in inherited neuropathies, it is of clinical importance to determine whether lower limb tremor is a contributing feature in CMT.

Consequently, the aim of the present study was to determine tremor characteristics in the upper and lower limbs of patients with CMT1A, to determine whether CMT-tremor is distinct from ET and to assess whether the presence of lower limb tremor was associated with imbalance.

## Methods

Patients with CMT1A were consecutively recruited from neuromuscular clinics in Sydney, Australia between July 2020 and August 2022. The diagnosis of CMT1A was based on the presence of a duplication in the *PMP22* gene (OMIM #118,220). Exclusion criteria included presence of an alternative medical condition that could explain tremor including medication-induced tremor. Prior to undertaking assessment, written informed consent was obtained from all participants and the study was ethically approved by the Western Sydney Local Health District Human Research Ethics Committee (2019/ETH08777/STE10638).

### Clinical assessments

All patients underwent comprehensive clinical assessment to characterize the clinical features of tremor and neuropathy. Tremor was assessed by the Tremor Research Group Essential Tremor Rating Assessment Scale (TETRAS), allowing categorization of tremor into rest, postural, and/or kinetic tremor, as well as grading tremor severity [19]. Disability associated with CMT neuropathy was calculated using the CMT Neuropathy Score, second version (CMTNS) [20]. Additionally, quality of life as it relates to tremor was measured with the Quality of Life in Essential Tremor (QUEST) questionnaire and reported as the QUEST Summary Index [21]. Upper and lower limb power was assessed by the Medical Research Council (MRC) sum score [22] and grip strength was assessed on both sides with a hand-held dynamometer. An overall assessment of lower limb sensorimotor function was calculated using the Neuropathy Impairment Score in the Lower Limbs (NIS-LL) scale [23], and gait speed was calculated with a timed 10-m walk test. Balance was measured using the Berg Balance Scale (BBS) [24], which assesses tasks such as standing from sitting, controlled descent to a chair, turning, transferring, reaching, and bending, scored from 0 (immobile) to 56 (normal) [24]. A score of 42, in combination with a history of imbalance, has a predicted probability of falling of 91% [25], thus a BBS score ≤ 42 was used to define patients with ‘poor’ balance, while a BBS > 42 indicated ‘good’ balance. Given the importance of ankle strength to balance [26, 27], the MRC scores of ankle dorsiflexion and plantarflexion were summated to give an ankle power score, with a minimum value of 0 (paralysis) and maximum value of 20 (full power). Vibration sense was also measured in the feet using a Rydel-Seiffer tuning fork, a graduated tuning fork that estimates vibration threshold by applying a numerical value, with normal being > 4 in the lower limbs [28].

### Tremor studies

Triaxial accelerometers and surface electromyography (EMG) were used to objectively characterize the neurophysiological features of tremor. Triaxial accelerometers were placed on the right index finger, positioned on the dorsal surface of the distal interphalangeal joint, and on the right knee, positioned over the patella. EMG electrodes were placed on the skin overlying abductor pollicis brevis, flexor carpi radialis, extensor carpi radialis, biceps brachii, triceps brachii, and deltoid muscles on the right upper limb. Lower limb recordings were taken from rectus femoris, biceps femoris, tibialis anterior, and medial gastrocnemius muscles using surface EMG, all on the right. Patients were seated in a large comfortable chair or instructed to stand, according to the conditions outlined in Table 1. In addition, a 500-g mass was applied during condition 7 to separate peripheral from central tremor, as tremor frequency reduces with increased mass in the former and remains stable in the latter [29].

**Table 1:** Conditions studied

	Upper limb conditions (seated)	Hand activity	Distraction
1	Hands at rest in participant’s lap, in mid-supination	Rest	Nil
2	Hands at rest in participant’s lap, in mid-supination	Rest	Mental
3	Forearms supported on armrest by participant’s side with hands hanging off the armrest	Rest	Nil
4	Arms hanging from the sides of the chair	Rest	Nil
5	Arms outstretched, hands pronated	Posture	Nil
6	Arms outstretched, hands pronated	Posture	Mental
7	Arms outstretched, hands pronated, 500 g weight	Posture	(Weight)
8	Arms outstretched, hands pronated, wrists extended	Posture	Nil
9	Hands in nose-targeting position	Posture	Nil
10	Finger-nose one per second, right hand	Right–kinetic Left–rest	Right–nil Left–motoric
11	Finger-nose one per second, left hand	Right–rest Left–kinetic	Right–motoric Left–nil

	Lower limb conditions	Hands	Eyes
12	Seated, legs held outstretched	Rest in lap	Open
13	Standing feet 16 cm apart	Rest by sides	Open
14	Standing feet 16 cm apart	Rest by sides	Closed
15	Standing feet 16 cm apart, arms outstretched	Posture	Open
16	Standing, feet together (0 cm apart)	Rest by sides	Open
17	Standing, feet together (0 cm apart)	Rest by sides	Closed

Legend: each participant underwent each of the listed conditions

All data were recorded using the Porti-system from TMSi (Oldenzaal, The Netherlands) for a duration of 30 s, with a sampling frequency of 2048 Hz. Data acquisition and tremor analysis were performed using a customized program written in MATLAB (Mathworks, Natick, Massachusetts, USA). To examine for muscular fatigue, raw tremor data were divided into two equal parts and the first and second halves were separately analyzed and compared.

### Tremor stability index

The tremor stability index (TSI) was calculated according to published methods [30]. In brief, triaxial accelerometry data from index finger recordings are analyzed for the first principal component and instantaneous frequencies are determined as the inverse of the interval between zero-crossings. The series of instantaneous frequencies are then used to calculate the interquartile range of the change in frequency, reported as the TSI. These values were compared to 26 pathological controls with upper limb tremor from a separate dataset obtained in our laboratory [31]. This included 12 patients with Essential Tremor (mean age, 59.8 ± 6.2; M:F, 6:6) and 6 with Essential Tremor Plus syndrome (mean age, 72.5 ± 2.7; M:F, 4:2).

### Posturography analysis

Balance was measured using a Kistler force platform (9260AA6, Kistler Group, Winterthur, Switzerland). Participants were assessed in conditions 12, 13, 15, and 16 as per Table 1, as well as tandem stance. Force in the anteroposterior, mediolateral, and vertical planes, as well as the center of pressure, were recorded for a duration of 15 s with a sampling frequency of 100 Hz. Participants were instructed to stand as still as possible and to fix on a visual target 1 m away in the “eyes open” condition. A trial was discarded if the patient required external support to maintain their balance, and the condition was repeated. If two trials were unsuccessful, the condition was abandoned. The data were analyzed using Fast Fourier Transform (FFT) to examine for a dominant spectral peak in force generated through the platform. The center of pressure parameters were calculated according to previously published techniques [32]. The velocity of sway was computed by taking the distance over time of each mini path, and power spectrum density analysis was applied to examine for the peak velocity of sway. The anteroposterior direction has been shown to be the most reliable variable [33], and so was the focus of the velocity analysis. All posturography assessments were performed blinded to the clinical findings and tremor analysis results.

### Nerve conduction studies

All patients underwent conventional neurophysiological assessment employing established methods [34]. Nerve conduction studies were performed on a single machine using Synergy software (Nicolet EDX by Natus, Pleasanton, CA, USA). In all cases, the right upper and lower limb were assessed. Motor nerve assessments included median, ulnar, and tibial nerves on the right, including F waves. Orthodromic sensory nerve conduction of the median, ulnar, and sural nerves, and an antidromic recording of the radial nerve were performed.

### Data analysis

Surface EMG was analyzed with FFT to define the peak tremor frequency and power, and principal component analysis was applied to the triaxial accelerometer data. To assess the role of fatigue, raw data were split into two equal recordings creating a first and second epoch and FFT was then computed. Statistical analysis was performed using R Statistical Software (version 3.6.3; R Foundation for Statistical Computing, Vienna, Austria). Data were compared using the student t-test for comparison of means if data were parametric, and the Mann–Whitney U test to compare medians if data were non-parametric. Pearson’s correlation coefficient was used to determine relationships between parametric variables and Spearman correlation if non-parametric. Linear regression modelling with backward elimination was used to predict tremor frequency based on clinical and neurophysiological findings. All data are expressed as mean ± standard error of the mean or median (interquartile range). A *P* value ≤ 0.050 (2-sided) was considered significant.

## Results

### Patients

In total, 17 patients with genetically confirmed CMT1A were recruited (6 males, 11 females). The mean age at assessment was 55.8 ± 2.8 years with mean BMI being 25.8 ± 1.1. The CMT neuropathy score was 18.1 ± 1.6 (range 8–30), indicating the presence of moderately severe neuropathy, with accompanying mild–moderate muscle weakness, moderate sensory loss, and imbalance (Table 2).

**Table 2:** Clinical features and tremor findings

Clinical features (*n* = 17)	Mean ± SEM
Age (years)	55.8 ± 2.8
BMI (kg/m^2^)	25.8 ± 1.1
CMTNS (< 10 = Mild; 11–20 = Moderate; > 20 = Severe)	18.1 ± 1.6
MRC Sum Score (normal 60)	54 ± 1
Grip strength (kilograms force)	18.4 ± 2.1
Rydel-Seiffer score, great toe (normal > 12)	0 (0–9)
10-m walk test (seconds)	8.1 ± 0.6
NIS-LL (normal 0, maximum 88)	38.5 ± 3.8
Berg Balance Scale (normal 56, poor balance ≤ 42)	40.7 ± 4.4
Tremor findings–upper limb posture (*n* = 12)	
Deltoid peak frequency (Hz)	9.5 ± 1.1
Biceps peak frequency (Hz)	9.1 ± 0.8
Extensor carpi radialis peak frequency (Hz)	9.1 ± 1.0
Abductor pollicis brevis peak frequency (Hz)	6.0 ± 0.4
Index finger accelerometry peak frequency (Hz)	6.7 ± 0.5
Tremor findings–lower limb posture (*n* = 6)	
Quadriceps peak frequency (Hz)	5.6 ± 0.4
Tibialis anterior peak frequency (Hz)	7.7 ± 0.4
Knee accelerometer peak frequency (Hz)	6.1 ± 1.1
Tremor findings–orthostatic (*n* = 6)	
Quadriceps peak frequency (Hz)	6.6 ± 1.7
Tibialis anterior peak frequency (Hz)	5.9 ± 1.4
Knee accelerometer peak frequency (Hz)	5.6 ± 0.8

Legend: Values are expressed as mean ± standard error of the mean or median (interquartile range). *BMI* body mass index, kg/m^2^ = kilograms per meter squared, *CMTNS* Charcot–Marie–Tooth Neuropathy Scale, *MRC* medical research council, *NIS-LL* neuropathy impairment score lower limbs, *Hz* Hertz

### Upper limb tremor

Postural and kinetic upper limb tremor was clinically evident in 65% of CMT1A patients, rated as mild as per the TETRAS score (range 3–16). Moderate-severe tremor was seen in 18% (TETRAS score range 31–34), which included tremor intruding into rest, although no patient had features of Parkinson’s disease. Quality of life was reduced in those with tremor as indicated by a significantly higher QUEST Summary Index (QSI_Tremor_ 9 (7–30), QSI_NoTremor_ 1 (0–5), P = 0.018).

In the upper limbs, a postural tremor of 9 Hz was observed in the deltoid, arm, and forearm regions, while the distal segments (abductor pollicis brevis muscle and index finger accelerometer) showed a peak at 6 Hz (Table 2, Supplementary Fig. 1a). The index finger peak frequency was significantly lower than the deltoid (*P* = 0.012) and arm (*P* = 0.011) regions (Supplementary Fig. 1c). Action tremor recorded from the index finger exhibited comparable peak frequencies to the postural tremor (Index finger_Posture_ 6.7 ± 0.5 Hz, Index finger_Action_ 6.3 ± 0.6 Hz, *P* = 0.783). When present, rest tremor frequency recorded at the index finger was 5.6 ± 0.4 Hz, comparable to index finger frequencies in posture and action (*P* = 0.789). There was no significant change in the tremor frequency with limb loading (Table 3). Additionally, peak spectral frequencies in the first and second half of the tremor recordings were comparable, arguing against an impact of limb fatigue (Table 3).

**Table 3:** Tremor characteristics

Weight loading–upper limb	No weight	Weighted	*P* value
Deltoid (Hz)	9.5 ± 1.1	9.3 ± 2.2	0.558
Arm (Hz)	9.1 ± 0.8	9.1 ± 0.9	0.614
Forearm (Hz)	9.1 ± 1.0	8.0 ± 1.3	0.465
Index (Hz)	6.7 ± 0.5	7.1 ± 0.7	0.798

	1st 15 s	2nd 15 s	*P* value
Effect of fatigue–upper limb			
Deltoid (Hz)	9.8 ± 0.9	9.5 ± 0.8	0.159
Arm (Hz)	8.5 ± 0.7	8.3 ± 0.7	0.137
Forearm (Hz)	7.9 ± 0.7	8.1 ± 0.8	0.595
Index (Hz)	7.3 ± 0.5	7.2 ± 0.6	0.776
Effect of fatigue–lower limb			
Quadriceps (Hz)	5.8 ± 0.5	5.6 ± 0.4	0.354
Tibialis anterior (Hz)	8.2 ± 0.6	7.7 ± 0.5	0.451
Knee accelerometry (Hz)	6.7 ± 0.5	6.7 ± 0.5	0.636

	Tremor	No Tremor	*P* value
Clinical features–upper limb			
Age (years)	56.9 ± 3.2	53.8 ± 6.0	0.660
BMI (kg/m^2^)	27.4 ± 0.8	22.9 ± 2.3	0.112
CMTNS (< 10 = Mild; 11–20 = Moderate; > 20 = Severe)	18.6 ± 2.4	17.0 ± 1.8	0.587
MRC Sum Score (normal 60)	54.0 ± 2.0	52.8 ± 1.6	0.663
Clinical features–lower limb			
Age (years)	54.5 ± 6.4	56.5 ± 3.0	0.779
BMI (kg/m^2^)	24.6 ± 1.7	26.4 ± 1.3	0.430
CMT neuropathy scale	16.8 ± 2.9	18.7 ± 2.0	0.604
MRC Sum Score (normal 60)	54.7 ± 2.1	53.0 ± 1.9	0.569
Ankle Power Score (normal 20)	17 [11–20]	17 [15–19]	0.758
NIS-LL (normal 0, maximum 88)	34.0 ± 6.7	40.9 ± 4.6	0.416
Rydel-Seiffer score (normal > 8)	0 [0–6]	0 [0–7]	0.822
10-m walk test (seconds)	7.5 ± 0.5	8.4 ± 1.0	0.454
Berg Balance Scale—orthostatic	53.0 (50.8–54.5)	48.0 (22.0–51.0)	0.226
Berg Balance Scale—postural	31.5 (16.5–46.5)	53.0 (49.0–55.5)	**0.030**

Legend: values are expressed as mean ± standard error of the mean, or median (interquartile range). Significant P values are signified by bold typeface. *Hz* Hertz, *BMI* body mass index, *kg/m*^2^ kilograms per meter squared, *CMT* Charcot–Marie–Tooth, *MRC* Medical Research Council, *NIS-LL* Neuropathy Impairment Score Lower Limbs, Berg Balance Scale normal score is 56, poor balance is categorized as ≤ 42. Charcot–Marie–Tooth Neuropathy Scale is categorised as mild (< 10), moderate (11–20), and severe (> 20)

Comparing CMT1A patients with and without tremor, there were no significant differences in clinical features including CMT neuropathy score and MRC sum score (Table 3) implying complex interactions between neuropathy findings and tremor generation not captured by these clinical scales. Linear regression modelling disclosed a significant correlation between peak frequency recorded in the forearm region and median nerve motor conduction velocity (beta = − 0.34, *P* = 0.021, average median conduction velocity 27.0 ± 2.9 m/s, range 11.4–42.5 m/s). The index finger accelerometry was also significantly correlated with median nerve velocity (beta = − 0.14, *P* = 0.004) and ulnar nerve F-wave latency (beta = 0.10, *P* = 0.017), suggesting that tremor frequency is associated with peripheral nerve conduction delay.

### Tremor stability index

**Fig. 1:**
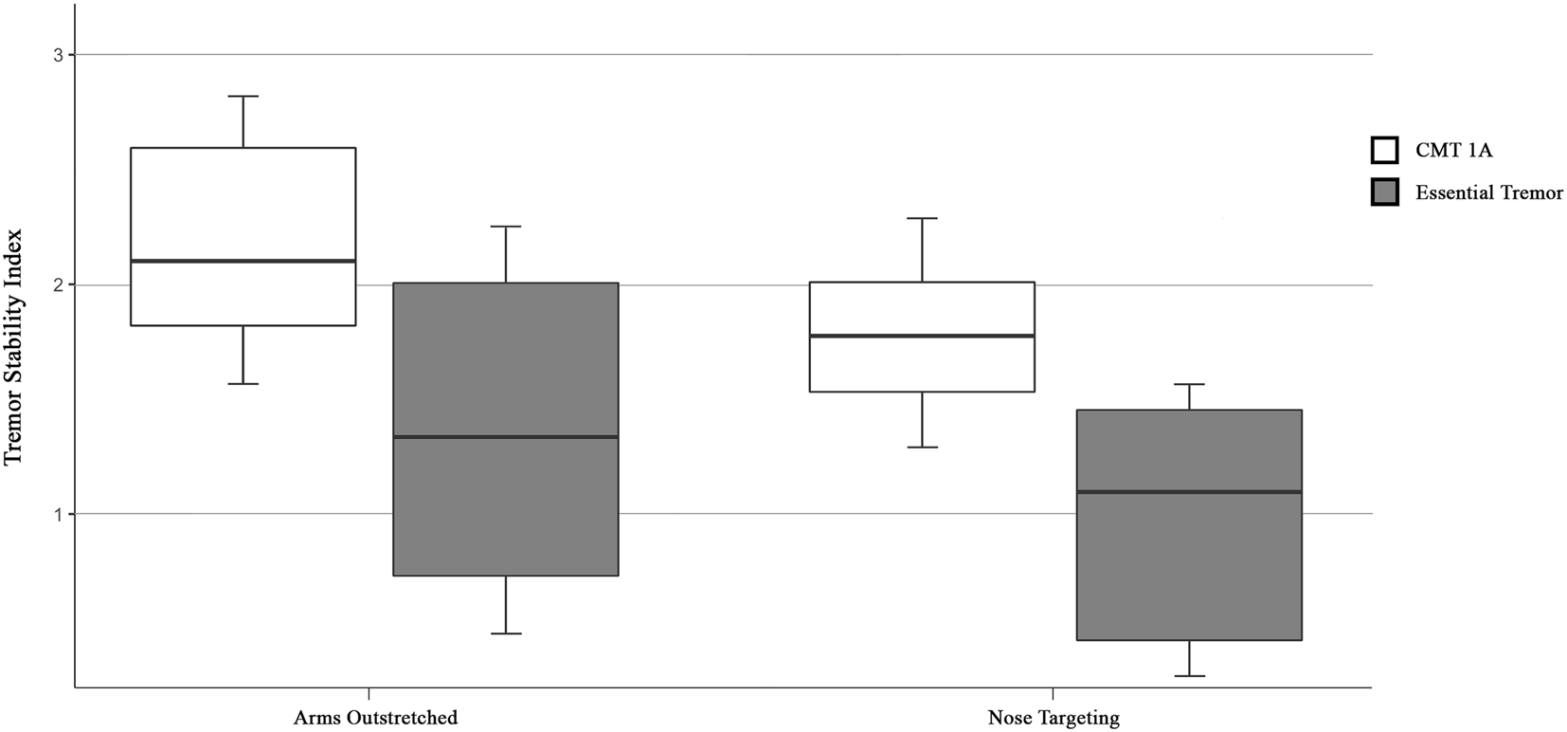
Tremor stability index. The tremor stability index was significantly higher in CMT1A patients in comparison to Essential Tremor patients in the arms outstretched and nose targeting positions

The tremor stability index was elevated in the “arms outstretched” and “nose-targeting” positions. The TSI was significantly higher in CMT1A patients when compared to ET (CMT_Arms Out_ 2.1 ± 0.2, ET_Arms Out_ 1.4 ± 0.2, *P* = 0.007; CMT_Nose_ 1.8 ± 0.2, ET_Nose_ 1.1 ± 0.2, *P* = 0.010, Fig. 1), but comparable to a recently published cohort with CIDP [10] (Arms Out 2.3 ± 0.1, *P* = 0.312, Nose 2.0 ± 0.2, *P* = 0.578).

### Lower limb tremor

Lower limb tremor was clinically evident in 35% of CMT1A patients with legs outstretched (Supplementary Fig. 1b). Subclinical orthostatic tremor was also observed in 35% of patients, and 12% of the cohort were found to have both postural and orthostatic lower limb tremor. Lower limb tremor was of lower frequency than upper limb tremor (Table 2), and there was no change in frequencies along the length of the lower limb (*P* = 0.135, Supplementary Fig. 1d, e). As in the upper limb, there was no difference between the first and second periods of tremor recordings (Table 2), arguing against a significant effect of fatigue.

A significant reduction in the Berg Balance Scale was evident in CMT1A patients that had a lower limb tremor (Tremor: 31.5 (16.5–46.5), No Tremor: 53 (49–55.5), *P* = 0.030, Table 3), while other demographic and clinical features were comparable (Table 3). Of relevance, MRC sum score, ankle power, and vibration sense were not significantly different between those with and without tremor (Table 3). Notably, lower limb nerve conduction studies were very reduced or absent in all but one patient with tremor, and all but two patients without tremor, which limited meaningful statistical analysis.

### Balance

**Fig. 2:**
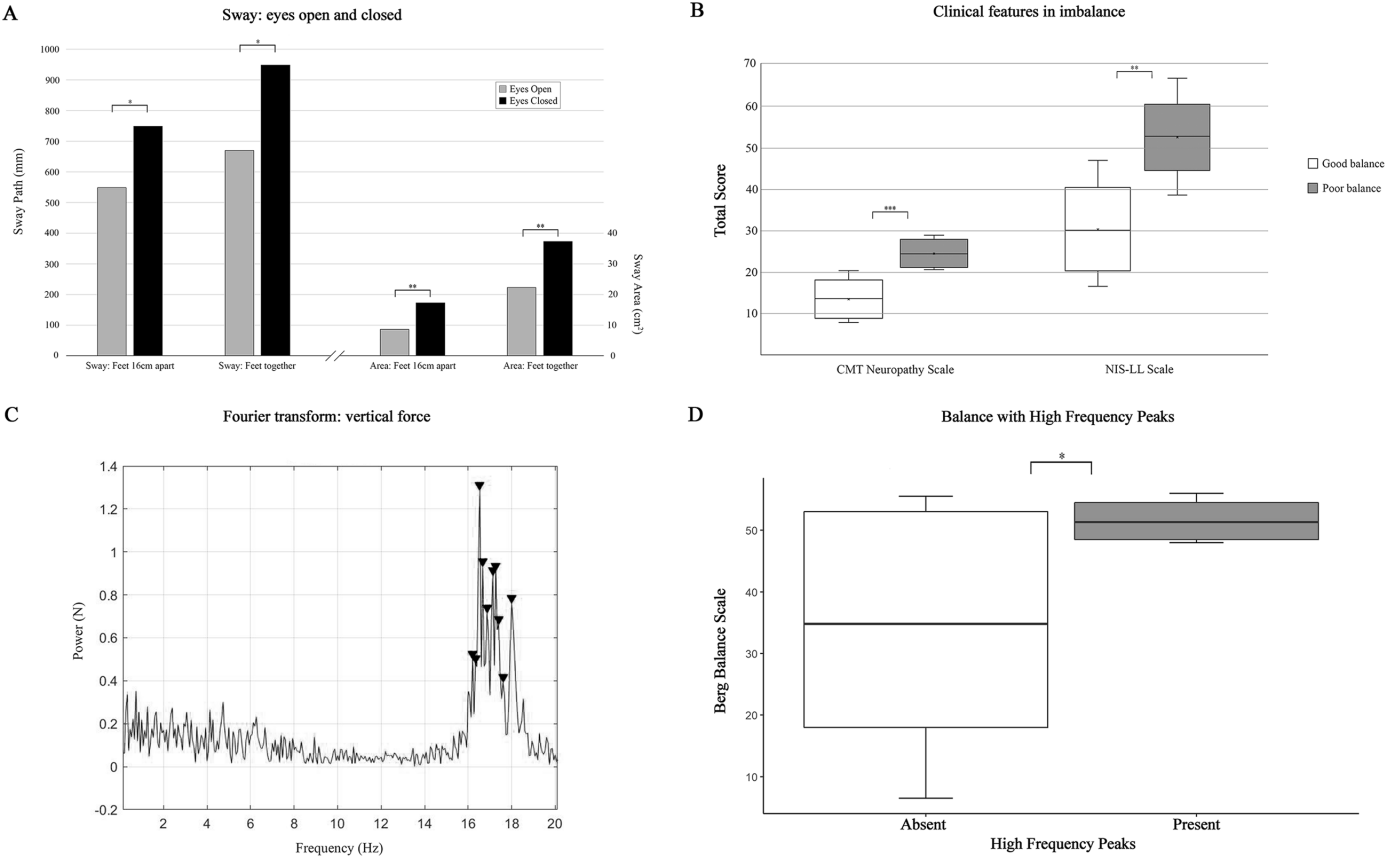
Posturography findings. Panel A: The sway path and sway area were significantly higher in the eyes closed conditions. Panel B: The CMT neuropathy scale and NIS-LL scales were both higher, indicating more severe neuropathy, in those with ‘poor’ balance. Panel C: A representative Fast Fourier Transform of the force applied through the platform in the vertical axis, demonstrating a peak around 16 Hz, which was only evident in those with ‘good’ balance. Panel D: Those with a high frequency peak as in Panel C had significantly higher Berg Balance Scale scores. *N* Newtons; *Hz* Hertz. mm millimeters; cm centimeters; * = *P* < 0.05; ** = *P* < 0.01, ***P < 0.001

The sway path and area were significantly elevated in CMT1A patients when assessed in the ‘eyes closed’ position (Fig. 2a), indicating a reliance on the visual system to maintain balance. The sway path was significantly correlated with the 10-m walk time (*R* = 0.54, *P* = 0.045) and the Berg Balance Score (BBS) (*R* = − 0.58, *P* = 0.030).

In CMT1A patients exhibiting ‘poor’ balance (BBS ≤ 42), the ankle power score was significantly reduced (‘good’ balance 20 (16–20), ‘poor’ balance 9 (4–15), *P* = 0.014). Further, the degree of neuropathy was more severe, as measured by clinical scales, in those with ‘poor’ balance (Fig. 2b).

The presence of postural lower limb tremor was associated with greater difficulty in completing the balance conditions. None of the patients with postural lower limb tremor could complete tandem stance while 75% of those without tremor could complete all conditions (*P* = 0.009). Multiple regression analysis found that the presence of lower limb postural tremor significantly associated with fewer balance conditions being completed (*β* = − 2.20, *SE* = 0.90, *t* = − 2.44, *p* = 0.031) independent of other variables.

In contrast, orthostatic tremor was more common in patients exhibiting ‘good’ balance (45%) than in ‘poor’ balance (17%), although this difference did not reach statistical significance (*P* = 0.235). Surprisingly, there were no significant differences in the sway path or sway area between patients with and without lower limb tremor (Supplementary Table 1).

The mean peak frequency of sway was 0.85 ± 0.1 Hz, similar to previously reported healthy controls [33], and did not differ between patients with and without lower limb tremor (Supplementary Table 1) nor in those with ‘good’ and ‘poor’ balance. The force exerted in the vertical plane was 4.3 ± 0.3 Hz across all conditions, similar to acquired demyelinating neuropathies [18]. Interestingly, a high frequency peak was observed in the vertical plane in 35% of patients, with median frequency of 17.1 Hz (15.8–17.4) (Fig. 2c), only evident in CMT1A patients with ‘good’ balance and none of the patients with ‘poor’ balance. The Berg Balance Scale was also significantly higher in those with a high frequency peak on posturography in comparison to those without a high frequency peak (Fig. 2d).

## Discussion

The present study established that postural upper limb tremor was a common clinical feature of CMT1A, with evidence of higher peak frequencies in the proximal (~ 9 Hz) and lower peak frequencies in the distal (~ 6 Hz) limb segments, unaffected by weight loading. The TSI was significantly higher when compared to essential tremor, and the upper limb tremor frequencies were significantly correlated with motor conduction velocity and F-wave latency. Taken together, the present findings suggest that CMT1A-related tremor is mediated by a complex interaction between central processes along with mistimed peripheral inputs, and appears to be distinct from essential tremor. Separately, postural and orthostatic lower limb tremor was evident in a third of CMT1A patients, with an absence of frequency change across the limb. Of relevance, the presence of lower limb postural tremor was associated with poor balance in CMT1A patients. The pathophysiological mechanism underlying CMT1A tremor and implications for management are further discussed.

### Pathophysiological mechanisms underlying CMT1A tremor.

Tremor observed in inherited neuropathies has been considered similar to essential tremor [11, 12]. The original descriptive studies were limited by a lack of confirmatory genetic testing, potentially resulting in heterogenous cohorts. Underscoring this notion are reports of neuropathic tremor in Roussy–Levy Syndrome [4], a condition now recognized as genotypically consistent with Charcot–Marie–Tooth disease type 1 [5, 6]. Additionally, previous studies examining CMT-tremor did not always include peripheral nerve conduction studies or tremor studies, thereby limiting conclusions [11, 35–37].

The present study assessed a well-phenotyped and genotyped cohort of CMT1A patients, utilizing objective clinical and neurophysiological measures to further determine the pathophysiological processes of CMT-tremor. While the peak tremor frequency was comparable to ET, the gradation of peak frequencies across the upper limb and significant differences in TSI indicated that CMT-tremor is a distinct entity, concordant with previous epidemiological studies [36, 38]. The frequency gradient in the upper limbs, along with significant correlations between tremor frequencies and median nerve conduction velocity and ulnar nerve F wave latencies, supports the possibility that CMT-tremor is mediated by a combination of mistimed peripheral inputs driving a potential central generator or oscillatory network. This is similar to tremor in inflammatory demyelinating neuropathies, also found to have correlations between peripheral nerve conduction and tremor findings [8, 10], as well as evidence of a tremor gradient in the upper limb [7, 10]. The significant increase in TSI provides additional support for a peripheral nerve contribution to tremor generation, as it was also elevated in acquired demyelinating neuropathies [10]. Mistimed peripheral inputs resulting from temporal dispersion of action potentials along demyelinated nerves could drive a postural tremor with inhomogeneous frequency, resulting in high beat-to-beat variability, reflected in the increased TSI in the present study.

A prominent role for central processes was also implicated in the present study. Upper limb weight-loading did not appreciably alter the CMT1A tremor frequency, a finding in keeping with previous studies on acquired demyelinating neuropathies [8, 10] and limb fatigue did not alter tremor characteristics, further supporting the importance of central mechanisms [16]. It remains unclear whether central structures are concurrently abnormal in demyelinating neuropathies, or are responding normally to distorted peripheral inputs. Further, the location of a central generator remains to be fully elucidated, although the ventral intermediate thalamic nucleus appears to be a good candidate [39, 40]. While cerebellar generators are implicated in acquired demyelinating neuropathic tremor [41, 42] such a mechanism has not been established in hereditary neuropathies [15]. Functional brain imaging studies may clarify the precise site of central tremor generators in CMT1A patients.

In addition to upper limb tremor, approximately a third of the CMT1A patients in the current study exhibited a postural or orthostatic lower limb tremor which was associated with poor balance. In contrast to upper limb tremor, there was no evidence for a proximal-to-distal reduction in the peak tremor frequencies, in keeping with findings in acquired demyelinating neuropathy related tremor [8]. Analysis of neurophysiological findings was limited in the lower limbs due to marked reduction or absence of responses in this primarily adult cohort. Future studies including younger patients with milder disease may further clarify whether there is a relationship between nerve conduction findings and lower limb tremor.

### Mechanisms underlying imbalance in CMT1A

The presence of lower limb tremor was independently associated with imbalance in the present cohort, in keeping with a recent cohort with CIDP [18]. Notably, ankle power and lower limb vibration sensation, which can independently cause imbalance [43], were not significantly different between those with and without tremor, further highlighting the independent association of lower limb tremor and imbalance. As lower limb tremor disrupts afferent signaling from muscle spindles and Golgi tendon organs [44], it is conceivable that lower limb tremor in the present cohort resulted in additional interruption of already dysfunctional somatosensory lower limb inputs, leading to greater imbalance. It should be noted that ankle weakness in the current cohort was associated with imbalance, in keeping with previous studies [27, 43], and there was a reliance on the visual system to maintain balance. Previous studies have reported that visual dependence of balance increases with age in CMT [45]; thus, the observed balance findings in the present study could also in part be explained by an older CMT cohort.

Of further relevance, a high frequency spectral peak in the vertical direction was recorded with posturography and associated with ‘good’ balance. The high frequency peak was similar to findings in acquired demyelinating neuropathy [18] and primary orthostatic tremor [46], and could represent an adaptive response to imbalance. A high frequency cortical drive to lower limb muscles coupled with increased cortical activity in the beta band (15–30 Hz) has been reported after balance perturbation in healthy controls [47, 48], and is postulated to be an adaptive response leading to joint stiffening, thereby increasing stability. Consequently, the observed high frequency spectral peak could represent a cortical drive to counteract imbalance in CMT1A. Interestingly, the presence of orthostatic tremor was associated with improved balance in CMT1A, which is contradictory to primary orthostatic tremor [44], but may in our cohort represent a similar adaptive mechanism.

The present study has some limitations. The cohort was relatively small which was influenced by the restrictions imposed during the COVID pandemic. This has the potential to limit statistical analysis and introduce false negatives. The age of the cohort and therefore, disease duration also meant that a proportion of patients had absent peripheral nerve conduction findings, further limiting analysis. Future studies with a larger cohort including a wider variety of ages will help to address these limitations.

In conclusion, the present study established postural and kinetic tremor as a common clinical feature in CMT1A, with a complex pathophysiology distinct from Essential Tremor. Postural and orthostatic lower limb tremor were also evident in a third of CMT1A patients, with the former associated with imbalance in combination with ankle weakness. Strategies aimed at improving ankle function and modulating lower limb postural tremor could prove therapeutically useful in CMT1A.

### Supplementary Information

Below is the link to the electronic supplementary material.

Supplementary file1 Supplementary Fig. 1 Tremor in CMT. Panel A: Representative traces of CMT-tremor with the arms outstretched. Panel B: Representative traces of CMT-tremor in the legs outstretched position. Panel C: Tremor frequency was lower in the distal segment of the upper limb compared with the proximal recordings. In the lower limbs, postural tremor (Panel D) and orthostatic tremor (Panel E) were not significantly different between the three regions of interest and were of lower frequency than tremor in the upper limbs (Panel C). Tremor power measured in Newtons (N) for electromyography (EMG) and Gravity (g) for accelerometry; tremor frequency measured in Hertz (Hz); ECR = extensor carpi radialis; APB = abductor pollicis brevis; Tib Ant = tibialis anterior; Index finger and knee data from accelerometry. ** denotes P < 0.05 (TIF 29392 KB)

## Data Availability

The data that support the findings of this study are available from the corresponding author upon reasonable request.
