# Supplementary figures and images for: Upper and lower limb tremor in Charcot–Marie–Tooth neuropathy type 1A and the implications for standing balance
Source: J Neurol. 2023 Dec 5;271(4):1776–86. doi: 10.1007/s00415-023-12124-z (PMC10972941; doi:10.1007/s00415-023-12124-z)

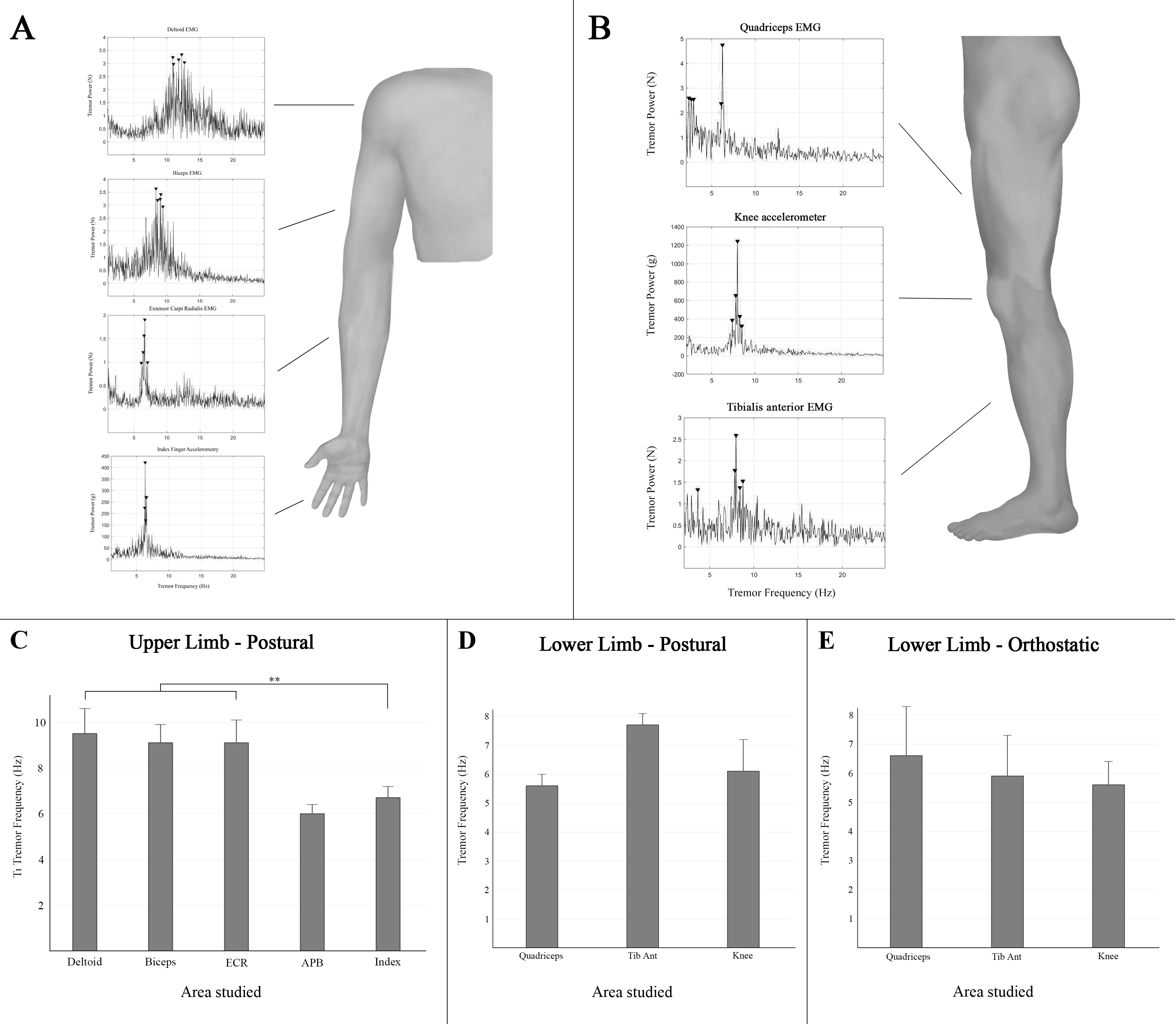

Supplement: Supplementary file 1 — Supplementary file1 Supplementary Fig. 1 Tremor in CMT. Panel A: Representative traces of CMT-tremor with the arms outstretched. Panel B: Representative traces of CMT-tremor in the legs outstretched position. Panel C: Tremor frequency was lower in the distal segment of the upper limb compared with the proximal recordings. In the lower limbs, postural tremor (Panel D) and orthostatic tremor (Panel E) were not significantly different between the three regions of interest and were of lower frequency than tremor in the upper limbs (Panel C). Tremor power measured in Newtons (N) for electromyography (EMG) and Gravity (g) for accelerometry; tremor frequency measured in Hertz (Hz); ECR = extensor carpi radialis; APB = abductor pollicis brevis; Tib Ant = tibialis anterior; Index finger and knee data from accelerometry. ** denotes P < 0.05 (TIF 29392 KB) [file 415_2023_12124_MOESM1_ESM.tif]
